# Perifocal edema is a risk factor for preoperative seizures in patients with meningioma WHO grade 2 and 3

**DOI:** 10.1007/s00701-024-06057-3

**Published:** 2024-04-06

**Authors:** Nico Teske, Nina C. Teske, Tobias Greve, Philipp Karschnia, Sabrina V. Kirchleitner, Patrick N. Harter, Robert Forbrig, Joerg-Christian Tonn, Christian Schichor, Annamaria Biczok

**Affiliations:** 1 Department of Neurosurgery, LMU University Hospital, LMU Munich, Marchioninistrasse 15, 81377 Munich, Germany; 2 German Cancer Consortium (DKTK), Partner Site Munich, Munich, Germany; 3 Center for Neuropathology and Prion Research, LMU University Hospital, LMU Munich, Munich, Germany; 4 Institute of Neuroradiology, LMU University Hospital, LMU Munich, Munich, Germany

**Keywords:** Meningioma, Atypical, Anaplastic, Epilepsy, Seizure, Edema

## Abstract

**Background:**

Patients with intracranial meningiomas frequently suffer from tumor-related seizures prior to resection, impacting patients’ quality of life. We aimed to elaborate on incidence and predictors for seizures in a patient cohort with meningiomas WHO grade 2 and 3.

**Methods:**

We retrospectively searched for patients with meningioma WHO grade 2 and 3 according to the 2021 WHO classification undergoing tumor resection. Clinical, histopathological and imaging findings were collected and correlated with preoperative seizure development. Tumor and edema volumes were quantified.

**Results:**

Ninety-five patients with a mean age of 59.5 ± 16.0 years were included. Most tumors (86/95, 90.5%) were classified as atypical meningioma WHO grade 2. Nine of 95 tumors (9.5%) corresponded to anaplastic meningiomas WHO grade 3, including six patients harboring TERT promoter mutations. Meningiomas were most frequently located at the convexity in 38/95 patients (40.0%). Twenty-eight of 95 patients (29.5%) experienced preoperative seizures. Peritumoral edema was detected in 62/95 patients (65.3%) with a median volume of 9 cm^3^ (IR: 0–54 cm^3^). Presence of peritumoral edema but not age, tumor localization, TERT promoter mutation, brain invasion or WHO grading was associated with incidence of preoperative seizures, as confirmed in multivariate analysis (OR: 6.61, 95% CI: 1.18, 58.12, *p* = *0.049). Postoperative freedom of seizures was achieved in 91/95 patients (95.8%).

**Conclusions:**

Preoperative seizures were frequently encountered in about every third patient with meningioma WHO grade 2 or 3. Patients presenting with peritumoral edema on preoperative imaging are at particular risk for developing tumor-related seizures. Tumor resection was highly effective in achieving seizure freedom.

**Supplementary Information:**

The online version contains supplementary material available at 10.1007/s00701-024-06057-3.

## Introduction

Meningiomas represent the most common primary brain tumors, accounting for up to 40% of all intracranial neoplasms [18]. Based on histological and molecular criteria, the 2021 World Health Organization (WHO) classification of CNS tumors defines three grades [16]. In this context, the current classification endorses the analysis of genetic alterations as their clinicopathological importance and prognostic relevance have emerged [16]. In particular, mutations of the telomerase reverse transcriptase (TERT) promotor region as well as homozygous deletion of CDKN2A/B were frequently observed in higher-grade meningiomas and associated with poor survival [1, 17, 21, 24], thus designating any of such tumors to CNS WHO grade 3 irrespective of its histologic subtype [16].

First-line treatment for the majority of symptomatic, enlarging or space-occupying meningiomas consists of microsurgical tumor resection with or without adjuvant radiation therapy (stereotactic radiosurgery or fractionated radiotherapy) [11]. Tumor resection aims to potentially cure patients, especially in WHO grade 1 tumors, but also serves to relief symptom burden in patients. In this regard, approximately one third of patients suffer from preoperative tumor-related seizures, significantly impacting patients’ quality of life, impairing cognitive function and inducing substantial morbidity [8]. Preclinical studies in malignant brain tumors have even suggested tumor-promoting effects of brain tumor-related epilepsy [28, 29], in line with large clinical studies demonstrating that tumor-related epilepsy is associated with worse survival in patients with glioblastoma or brain metastases [17]. The implications of tumor-related epilepsy on the oncological outcome in meningiomas remain to be defined. Studies predominantly investigating meningiomas WHO grade 1 have found male sex, absence of headache, tumor localization and peritumoral edema to be independent risk factors for tumor-related epilepsy [8, 12, 19]. Histologically, brain invasion was found to be associated with an increased risk for preoperative seizures [12]. The pathogenesis of tumor-related epilepsy seems to be multifactorial including effects of the peritumoral vasogenic edema and morphologic changes of the peritumoral neocortex [9]. Following tumor removal, most patients achieve long-term freedom of seizures. However, a distinct subset of patients still suffers from late or persisting postoperative seizures in particular thus posing a challenge for antiepileptic drug (AED) management [6, 8]. Here, preoperative tumor-related seizures, preoperative edema and postoperative surgery-induced changes were found to be risk factors for postoperative epilepsy [14].

Accurate prediction and identification of risk factors for tumor-related epilepsy in patients with meningioma remain crucial to improve treatment strategies and guide clinical management, impacting recommendations for prophylactic AED treatment as well as postoperative neurologic follow-up. Such studies investigating patients with meningiomas WHO grade 2 and 3 based on histologic and molecular features defined by the WHO 2021 classification are currently still missing. In this single-center study, we describe a retrospective cohort of 102 patients with meningioma CNS WHO grade 2 and 3 (WHO 2021 classification), undergoing microsurgical tumor resection. We aim to (1) identify risk factors for tumor-related seizures, (2) compare functional outcomes and survival between patients with and without preoperative seizures and (3) discuss implications for patient management.

## Methods

### Study population and trial design

**Fig. 1:**
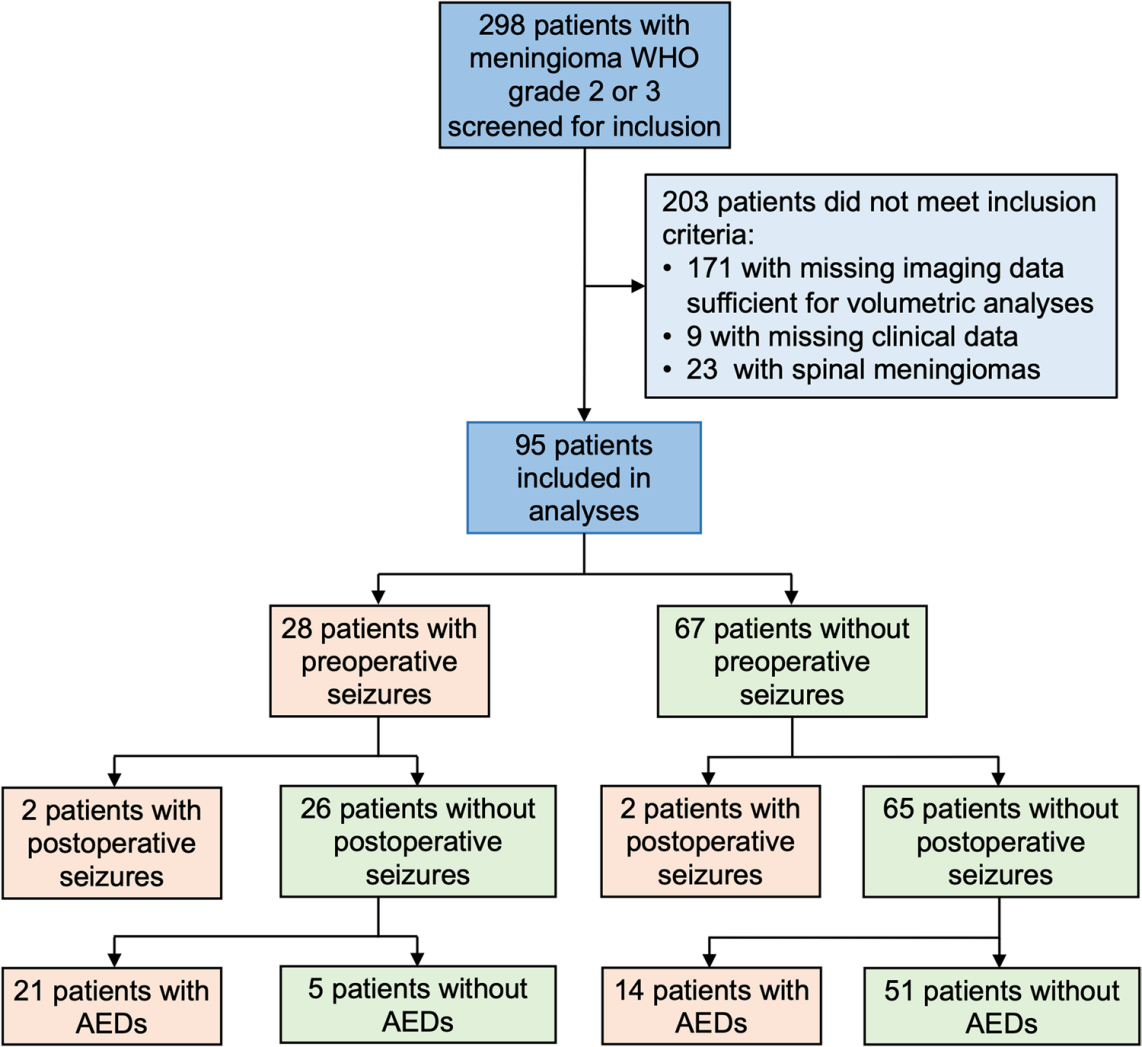
Consort diagram for patient selection. Schematic representation reporting patient screening, patient selection for analyses and follow-up for the entire patient cohort with meningioma WHO grade 2 and 3 undergoing microsurgical tumor resection at the Center for Neuro-Oncology at the Ludwig-Maximilians-University Munich (*n* = 95)

The study was designed as a single-center retrospective analysis of our institutional database within the framework of the Center for Neuro-Oncology at the Ludwig-Maximilians-University. Study protocol and design were approved by the Institutional Review Board of the Ludwig-Maximilians-University in Munich, Germany (AZ 18–837), and informed patient consent was obtained. Patients presenting with meningioma WHO grade 2 or 3 undergoing microsurgical tumor resection between 2013 and 2023 were screened for inclusion. Data cutoff for the study cohort was December 1, 2023. Inclusion criteria for patient selection consisted of (1) age ≥ 18 years, (2) tissue-based diagnosis of intracranial meningioma CNS WHO grade 2 or 3 according to the 2021 WHO classification including TERT promotor mutation status whenever available [16], (3) first-line treatment consisting of microsurgical tumor resection and (4) pre- and postoperative MRI available for review and sufficient for detailed volumetric analyses (Fig. 1). No further inclusion criteria were applied, and patients were treated consecutively at our institution thus avoiding introduction of confounders. All tumor resections were performed or supervised by two senior neurosurgeons with long lasting experience in meningioma surgery (J.C.T, C.S.). Subgroup analyses were performed for all patients where TERT promotor status was available. Clinical information including demographics, histopathologic findings, treatment specifics including perioperative AED management, imaging and outcome data were collected. Multidisciplinary tumor board recommendations and patient preference formed the basis for treatment decisions.

### Seizure management

Preoperative seizures were diagnosed clinically and with electroencephalography (EEG). Patients were treated with AEDs preoperatively in case of already diagnosed seizures or started on AEDs prophylactically at the discretion of the treating physician in case of extensive mass effect. AED selection was based on current guidelines for treating focal or generalized seizures considering potential drug-drug interactions, toxicities, and efficacy in tumor-related epilepsy. Patients presenting with seizures were followed postoperatively via repeated EEG to guide therapy. Outcome of seizure control after tumor resection was documented according to the Engel classification [7] and the *International League Against Epilepsy* (ILAE) classification of outcome following surgery [27].

### Magnetic resonance imaging

Standardized brain tumor imaging protocols including thin-sliced pre-contrast T1-weighted sequences, contrast-enhancing (CE) T1-weighted sequences and T2-weighted sequences were applied to ensure detailed volumetric analyses in all patients. Follow-up imaging and surveillance scans were performed according to current guidelines every 1–2 years or in case of clinical deterioration [11]. Definitions of residual tumor remnants, tumor recurrence or progression on MRI were based on contemporary guidelines of the Response Assessment in Neuro-Oncology (RANO) Working Group [13]. In detail, tumor remnants were defined as residual bidimensional CE lesions in the region of the resection cavity and denoted as ‘subtotal resection’. Accordingly, ‘gross total resection’ was defined as no residual CE lesion on postoperative MRI. Any progressive contrast enhancement on follow-up imaging compared to baseline postoperative MRI was denoted as tumor progression. Tumor recurrence was defined as any new contrast enhancement on follow-up imaging in cases where gross total resection was achieved according to baseline postoperative MRI. Preoperative CE tumor and edema volumes were quantified using a commercially available segmentation software (BrainLab® Elements; Munich). Absolute tumor and edema volumes (cm^3^) were recorded, and total volume (tumor + edema volume) was calculated. An identical set of ten randomly assigned preoperative MRIs was analyzed by two raters independently to allow quantification of an inter-rater variability.

### Neuropathological diagnosis

Histopathologic diagnosis was based on the WHO 2021 classification of CNS tumors from tissue sampled during tumor resection [16]. Tumor samples initially analyzed before 2021 underwent re-classification according to the current WHO 2021 classification. For TERT promoter mutation status, Sanger sequencing was performed for the two hot-spot mutations C250T and C228T located upstream of the TERT gene, as previously described [1, 26]. In case of extended molecular diagnostics, next-generation sequencing or DNA methylation-based diagnostics were performed whenever deemed clinically necessary [2].

### Statistical analysis

Clinical, histopathologic and imaging characteristics were summarized in total and for patients with and without preoperative seizures, respectively. For descriptive statistics, categorical variables are expressed in absolute numbers and percentages. Visual inspection of frequency histograms and the D’Agostino-Pearson omnibus normality test were used to test for normal distribution and equal variance in continuous data. Normally distributed numerical data are described as mean ± standard error of the mean, and range is given. Otherwise, data are presented as median ± interquartile range (IR).

Associations between two or more variables were analyzed using the chi-squared test and Fisher’s exact test. In case of parametric data, differences between two groups were assessed with the unpaired Student’s *t* test. For nonparametric variables, the Mann–Whitney *U* test was calculated. Multivariate logistic regression was used to evaluate predictors of preoperative seizures, adjusted for statistically significant and clinically meaningful risk factors, and odds ratios (OR) with their respective 95%-confidence intervals (CI) were calculated. For survival analyses, Kaplan–Meier survival estimates were generated, and log-rank tests were calculated to compare overall survival and recurrence-free survival between patient cohorts. Patients were followed until data cutoff (December 1, 2023) or death. Individuals with a follow-up time of less than 1 month were excluded from survival analyses. Patients lost to follow-up were censored at day of last follow-up. Date of diagnosis was set as date of tumor resection. Overall survival was defined as the interval from diagnosis to death from any cause, and recurrence-free survival was defined as the interval from diagnosis to recurrence. Inter-rater agreement on volumetrics was evaluated by determining the intraclass correlation coefficient (ICC; reliability: < 0.5: poor, 0.5–0.75: moderate, 0.75–0.9: good, > 0.9: excellent)[15]. Statistical analyses were performed using Prism statistical software (Prism 10.0; GraphPad Software Inc., San Diego, CA, USA) and Stata statistical software (Stata 17. 0; StataCorp LLC., College Station, TX, USA). The significance level was set at *p* ≤ 0.05.

## Results

### Patient characteristics

Among 298 screened patients with meningioma WHO grade 2 or 3 undergoing microsurgical tumor resection at our institution between 2013 and 2023, 203 were excluded due to missing imaging or clinical data or due to spinal tumor localization (Fig. 1). Ninety-five patients were included for analyses with a mean age at diagnosis of 59.5 ± 16.0 years (range: 21–86 years) and a male-to-female ratio of 1:2.0. At time of diagnosis, patients most often presented with focal or generalized seizures (28/95 patients, 29.5%) and were treated with AEDs. Six of 67 patients (9.0%) without preoperative seizures received prophylactic AED treatment at the discretion of the treating physician mostly due to extensive mass effect with the risk for herniation. Cranial nerve palsies and cognitive deficits were frequently encountered in 17/95 patients (17.9%) and 14/95 patients (14.7%), respectively. Other symptoms included headaches and sensorimotor deficits (Table 1). In contrast, asymptomatic cases with tumors diagnosed on the basis of incidental imaging findings were observed in 20/95 patients (21.1%).

**Table 1:** Patient characteristics

		Preoperative seizures	No preoperative seizures	Total	*p* value
Overall, *n* (%)		28 (29.5%)	67 (70.5%)	95 (100%)	
Age at diagnosis, mean (SD)		62.7 ± 12.7	58.1 ± 17.1	59.5 ± 16.0	0.209
Sex, *n* (%)	Female	18 (64.3%)	45 (67.2%)	63 (66.3%)	0.815
	Male	10 (35.7%)	22 (32.8%)	32 (33.7%)	
Clinical characteristics					
Additional symptoms, *n* (%)	Cranial nerve palsy	2 (7.1%)	15 (22.4%)	17 (17.9%)	*0.001
	Cognitive deficits	3 (10.7%)	11 (16.4%)	14 (14.7%)	
	Headache	0 (0%)	13 (19.4%)	13 (13.7%)	
	Sensorimotor deficits	4 (14.3%)	9 (13.4%)	13 (13.7%)	
	Aphasia	3 (10.7%)	3 (4.5%)	6 (6.3%)	
	Incidental	0 (0%)	20 (29.9%)	20 (21.1%)	
postOP (transient) deficits, *n* (%)	Yes	2 (7,1%)	13 (19.4%)	15 (15.8%)	0.217
	No	26 (92.9%)	54 (80.6%)	80 (84.2%)	
MRI characteristics					
Location	Convexity	13 (46.4%)	25 (37.3%)	38 (40.0%)	0.473
	Skull base	10 (35.7%)	25 (37.3%)	35 (36.8%)	
	Parafalcine	5 (17.8%)	12 (17.9%)	17 (17.9%)	
	Intraventricular	0 (0%)	5 (7.5%)	5 (5.3%)	
Extent of resection, *n* (%)	Gross total resection	19 (67.9%)	44 (65.7%)	63 (66.3%)	0.999
	Subtotal resection	9 (32.1%)	23 (34.3%)	32 (33.7%)	
Contrast enhancement, *n* (%)	homogenous	15 (53.6%)	34 (50.8%)	49 (51.6%)	0.826
	heterogenous	13 (46.4%)	33 (49.3%)	46 (48.4%)	
Edema, *n* (%)	Present	26 (92.9%)	36 (52.9%)	62 (65.3%)	*0.001
	Absent	2 (7.1%)	32 (47.1%)	34 (35.8%)	
Volumetric analyses, median cm^3^ (IR)	Tumor volume	33 (16–84)	28 (15–66)	31 (15–66)	0.471
	Edema volume	49 (9–93)	0 (0–38)	9 (0–54)	*0.001
	Total volume	94 (41–156)	44 (16–125)	68 (22–139)	*0.005
Neuropathology					
WHO grade 2021, *n* (%)	Grade 2	26 (92.9%)	60 (89.6%)	86 (90.5%)	0.999
	Grade 3	2 (7.1%)	7 (10.4%)	9 (9.5%)	
TERT promotor, *n* (%)	Wildtype	22 (78.6%)	39 (58.2%)	61 (64.2%)	0.656
	Mutated	1 (3.6%)	5 (7.5%)	6 (6.3%)	
	n.a	5 (17.9%)	23 (34.3%)	28 (29.5%)	
Brain invasion, *n* (%)	Present	12 (42.9%)	22 (32.8%)	34 (35.8%)	0.360
	Absent	16 (57.1%)	45 (67.2%)	61 (64.2%)	
Adjuvant treatment					
Radiotherapy, *n* (%)	Yes	9 (32.1%)	28 (41.8%)	37 (38.9%)	0.490
	No	19 (67.9%)	39 (58.2%)	58 (61.1%)	

Characteristics are given for all patients with intracranial meningioma WHO 2021 grade 2 or 3 (*n* = 95), patients presenting with preoperative seizures (*n* = 28) or without preoperative seizures (*n* = 67). The significance level was set at *p* ≤ 0.05, indicated by *

*n* count, *postOP* postoperative, *SD* standard deviation

### Imaging and extent of resection

**Fig. 2:**
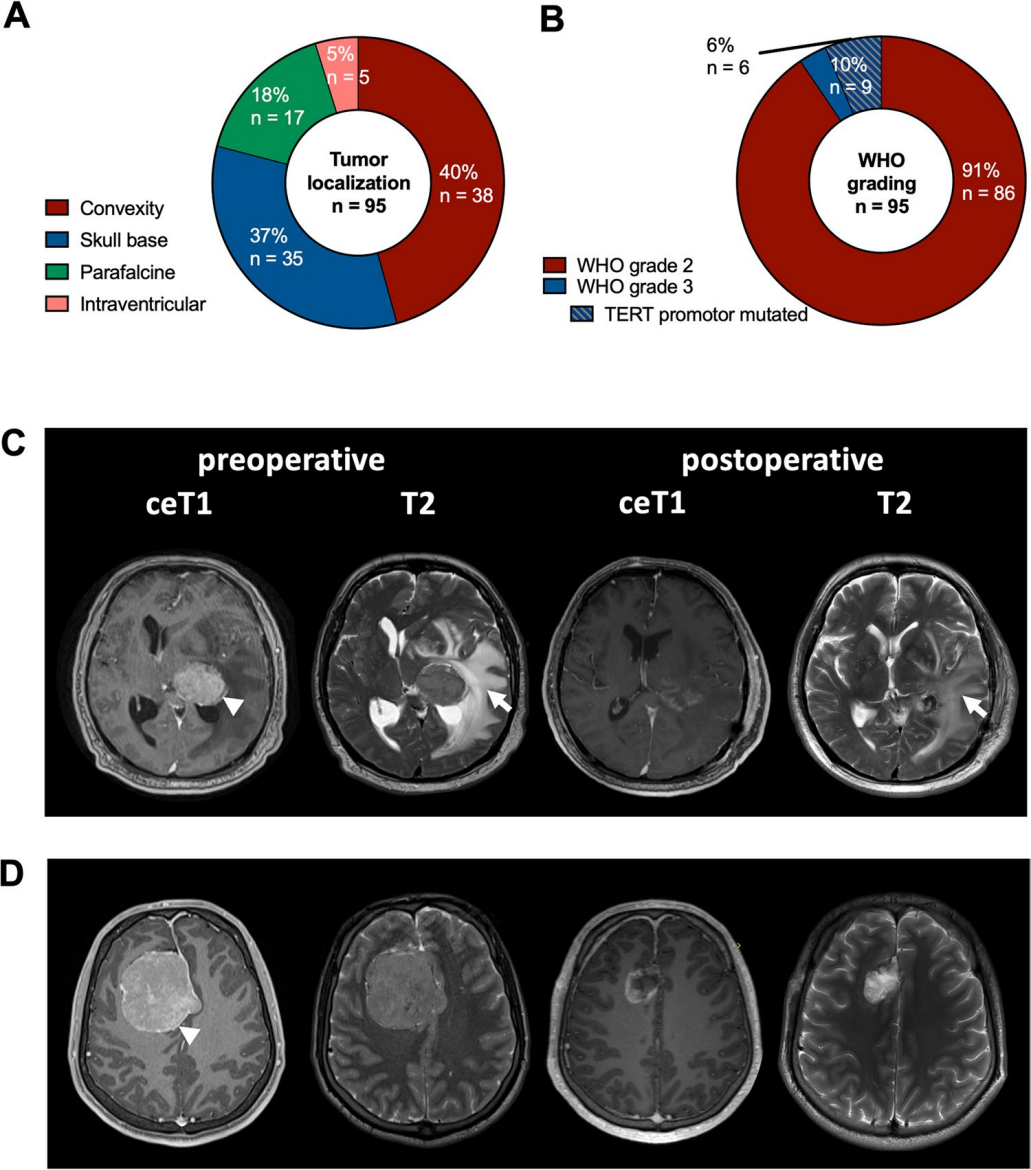
Tumor characteristics and case examples. **A** Distribution of tumor localization across the entire study cohort (*n* = 95). **B** Distribution of tumor grading according to the 2021 WHO classification including TERT promotor mutation status in the entire study cohort (*n* = 95). **C** Preoperative and postoperative axial contrast-enhancing T1-weighted MRI demonstrating homogenous contrast enhancement of a left-sided intraventricular meningioma WHO grade 2 (arrowhead) with no apparent residual tumor after resection. T2-weighted MRI sequences showing extensive preoperative perifocal edema slowly decreasing on postoperative imaging. **D** Preoperative axial contrast-enhancing T1-weighted MRI demonstrating homogenous contrast-enhancement of a right-hemispheric frontal parafalcine meningioma WHO grade 2 (arrowhead) without perifocal edema on T2-weighted MRI. Postoperative MRI showing complete tumor resection

Meningiomas were most commonly located at the convexity (38/95 patients, 40.0%), followed by the skull base (35/95 patients, 36.8%), and the parafalcine/parasagittal region (17/95 patients, 17.9%; Fig. 2A). In five of 102 patients (5.3%), imaging revealed intraventricular meningioma localization. On postcontrast T1-weighted imaging, all tumors presented with contrast enhancement while almost half of the tumors (46/95 tumors, 48.4%) demonstrated heterogenous contrast enhancement. Edema was present in 62/95 patients (65.3%). Median preoperative tumor volume on contrast-enhancing T1-weighted imaging was 31 cm^3^ (IR: 15–66 cm^3^, range: 2–223 cm^3^) with a corresponding median preoperative edema volume on T2-weighted imaging of 9 cm^3^ (IR: 0–54 cm^3^, range: 0–204 cm^3^) in the entire patient cohort. Preoperative tumor and edema volume did not correlate (*p* = 0.806). Gross total tumor resection was achieved in 63/95 patients (66.3%). We found excellent inter-rater agreement for segmentations of tumor volumes (ICC 0.990) and edema volumes (ICC: 0.996).

### Neuropathology and adjuvant therapies

The majority of tumors were classified as atypical meningiomas WHO grade 2 (86/95 tumors 90.5%). The remaining nine of 95 tumors (9.5%) corresponded to anaplastic meningiomas WHO grade 3 (Fig. 2B). TERT promotor mutation status was available in 67/95 patients (70.5%), revealing TERT promotor mutations in six of 67 patients (9.0%) and leading to a re-classification as anaplastic meningioma WHO grade 3 in three cases. Brain invasion was present in 34/95 patients (35.8%) and was independent of tumor volume. However, edema volume was higher in invasive tumors in comparison to noninvasive tumors (27.3 *versus* 4.8 cm^3^ edema volume, *p = 0.032).

Most patients, all of them diagnosed with meningioma WHO grade 2, were postoperatively followed via a watch-and-wait approach with surveillance scans (58/95 patients, 61.1%). Based on interdisciplinary tumor board recommendations, adjuvant radiotherapy was administered in 37/95 patients (38.9%), due to diagnosis of an anaplastic meningioma WHO grade 3 or in case of subtotal tumor resection in meningiomas WHO grade 2.

### Predictors for preoperative seizures

**Fig. 3:**
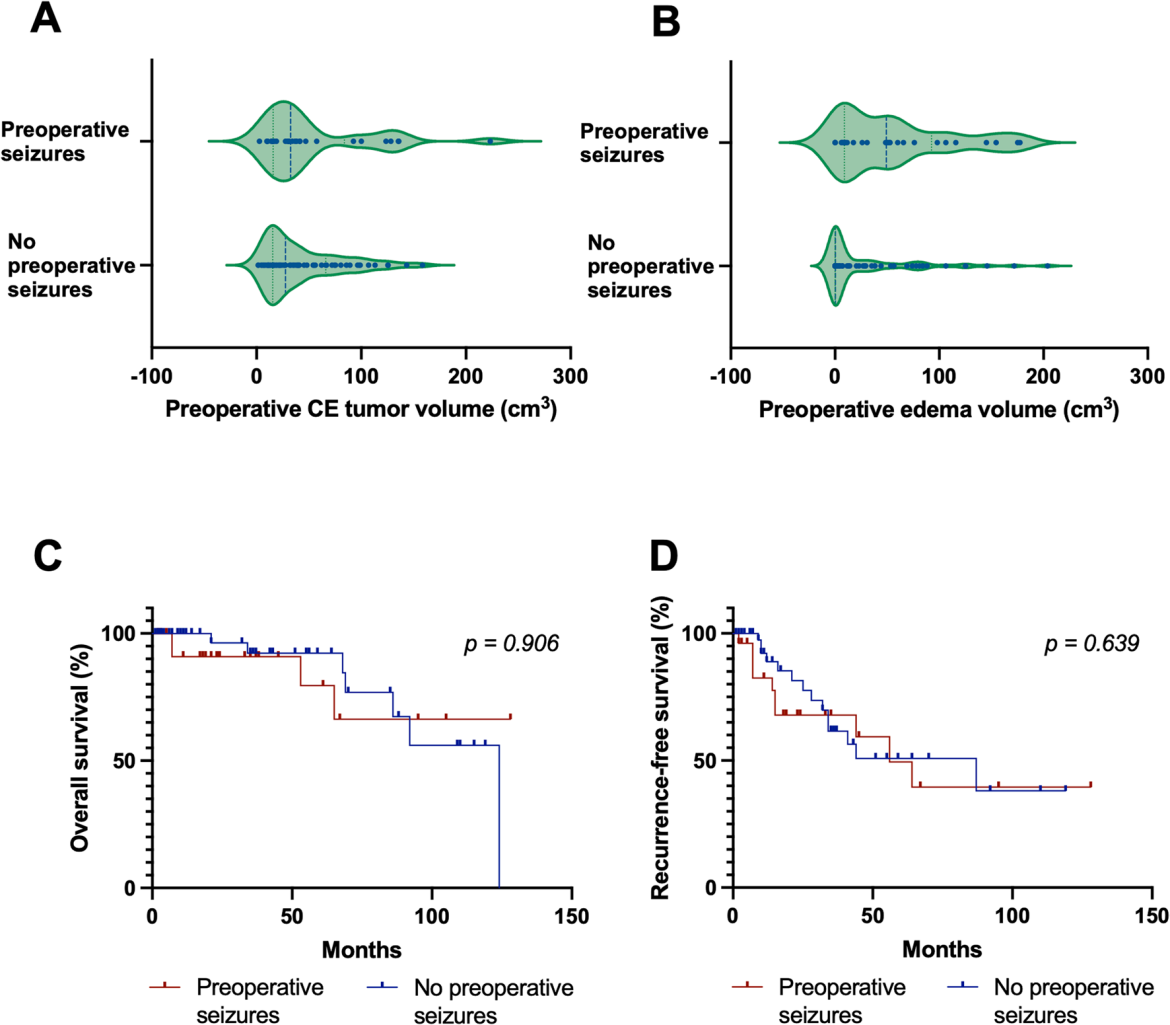
Survival depending on preoperative seizures. **A–B** Preoperative tumor (**A**) and edema (**B**) volume in patients presenting with and without preoperative seizures. **C–D** Kaplan–Meier estimates of overall survival (**C**) and recurrence-free-survival (**D**) in the entire study cohort (*n* = 95). Curves are given for patients with (red; *n* = 28) and without (blue; *n* = 67) preoperative seizures. Tick marks indicate censored patients. *Abbreviations: CE* contrast-enhancing

The entire study cohort was divided into two subgroups consisting of (1) patients presenting with tumor-related preoperative seizures (28/95 patients, 29.5%) and (2) patients without tumor-related preoperative seizures (67/95 patients, 70.5%, Fig. 2C, D). Clinical, imaging and histopathological baseline characteristics before microsurgical tumor resection were compared. Age and male-to-female ratio did not differ between both subgroups (age: *p* = 0.209, sex: *p* = 0.815, respectively). As expected, the spectrum of preoperative symptom burden differed between both subgroups as patients without preoperative seizures more commonly presented with different symptoms such as headaches, cranial nerve involvement and cognitive impairment, or with incidental imaging findings (*p = 0.001; Table 1). In our cohort, the risk for developing preoperative seizures was independent of anatomical tumor localization (*p* = 0.473). Notably, presence of edema (26/28 [92.9%] *versus* 36/67 [52.9%], *p = 0.001), higher edema volume (49.3 *versus* 0.3 cm^3^ edema volume, *p = 0.001), as well as total volume (tumor + edema; 94.4 *versus* 43.8 cm^3^ total volume, *p = 0.005) but not tumor volume itself were associated with preoperative seizures (Fig. 3A, B). In regard to histopathological characteristics, neither WHO grading, TERT promotor mutation status nor presence of brain invasion differed between both groups (*p* = 0.999, *p* = 0.656, *p* = 0.360, respectively); however, we cannot exclude bias due to small sample sizes.

Additionally, multivariate logistic regression was performed to control for potential confounding effects. Statistically significant risk factors for preoperative seizures in the univariate analysis and already known risk factors based on previous publications were included. Our multivariate model included age, sex, tumor localization, presence of edema, volumetric analyses and histopathological variables (WHO, TERT promotor mutation status, brain invasion). Here, only presence of edema on preoperative MRI remained an independent risk factor for preoperative seizures in patients with meningioma WHO grade 2 or 3 (OR 6.61, 95%-CI 1.18, 58.12, *p = 0.049, Table 2).

**Table 2:** Multivariate logistic regression analysis for predicting preoperative seizures

Risk factor	Odds ratio	95% CI	*p* value
Age at diagnosis, years	1.04	0.99, 1.09	0.151
Sex			0.738
Female (R)	1		
Male	1.29	0.28, 5.87	
Location, *n* (%)			
Skull base (R)	1		
Convexity	1.55	0.35, 7.25	0.563
Parafalcine	2.81	0.43, 19.66	0.281
Edema			
Absent (R)	1		
Present	**6.61**	**1.18, 58.12**	*0.049
Edema volume, cm^3^	1.01	0.99, 1.02	0.312
Tumor volume, cm^3^	1.00	0.99, 1.01	0.604
WHO grade 2021			
Grade 2 (R)	1		
Grade 3	1.30	0.02, 80.86	0.899
TERT promotor			
Wildtype (R)	1		
Mutated	0.14	0.00, 13.35	0.378
Brain invasion, *n* (%)			
Absent (R)	1		
Present	1.24	0.26, 5.79	0.785

Multivariate logistic regression for predicting preoperative seizures, adjusted for statistically significant and clinically meaningful risk factors in patients with intracranial meningioma WHO 2021 grade 2 or 3. Five patients with intraventricular localization were excluded due to multicollinearity (*n* = 90). The significance level was set at *p* ≤ 0.05, as indicated by *

*R* reference

Moreover, predictors for preoperative seizures were assessed in the subgroup of meningiomas WHO grade 2 and 3 with available TERT promotor mutation (*n* = 67). For this end, patients were divided into (1) patients presenting with tumor-related preoperative seizures (23/67 patients, 34.3%) and (2) patients without tumor-related preoperative seizures (44/67 patients, 65.7%) as described before. Here, clinical baseline characteristics did not differ between patients with and without preoperative seizures except for spectrum of symptom burden similar to our entire study cohort (Suppl. Table 1). In univariate analyses, presence of edema (21/23 [91.3%] *versus* 26/44 [59.1%] patients, *p = 0.010), higher edema volume (52.5 *versus* 4.5 cm^3^ edema volume, *p = 0.002) as well as total volume (tumor + edema; 106.7 *versus* 44.8 cm^3^ total volume, *p = 0.011), but not tumor volume itself were associated with preoperative seizures corresponding to our findings in the entire study cohort. Moreover, multivariate logistic regression analyses were performed in this subgroup using the multivariate model described above. Again, only presence of edema remained an independent risk factor for preoperative seizures in patients with meningioma WHO grade 2 or 3 and available TERT promotor mutation status (OR 6.61, 95%-CI 1.18, 58.12, *p = 0.049, Suppl. Table 2).

### Postoperative seizure control and long-term survival

Tumor resection was generally well-tolerated with 15/95 patients (15.8%) experiencing mild-to-moderate new focal neurological deficits which were often transient in nature. Only four of 95 patients (4.2%) developed postoperative seizures within a median follow-up time of 21 months (range: 1–128 months) in the entire patient cohort. Two of those patients experienced preoperative seizures; the other two had new-onset seizures after surgery, respectively. In detail, two patients developed simple focal seizures during the first year after surgery and were classified as ILAE class 3 and Engel class 1B at that time. During follow-up, one of those patients remained seizure free for over 1 year leading to a ILAE class 1 reclassification at the end of follow-up. Another patient initially developed two disabling generalized tonic–clonic seizures but remained free of seizures for over 2 years after adjustment of AED management, leading to a ILAE class 1 and Engel class IC at the end of follow-up. Finally, one patient who presented with acute seizure clusters preoperatively developed a significant and worthwhile reduction in seizure activity postoperatively corresponding to ILAE class 4 and Engel class IIIA. The remaining 91/95 patients (95.8%) were completely seizure free after tumor resection corresponding to class IA of the Engel classification and class 1 on the ILAE outcome scale. Thirty-five of those 91 patients (38.5%) were treated with AEDs postoperatively. Preoperative seizures were not significantly associated with postoperative seizures in our cohort (*p* = 0.358); however, sample size was limited with only four patients experiencing postoperative seizures. Also, gross total tumor resection was not associated with reduced postoperative seizure rates (*p* = 0.999).

Overall, 24/95 patients (25.3%) suffered from tumor progression or recurrence, and 11/95 patients (11.6%) eventually died of tumor progression within a median follow-up time of 21 months (range: 1–128 months). Median overall survival (OS) was 124 months (range: 1–128 months), and median recurrence-free survival (RFS) was 56 months (range: 1–128 months). Next, we evaluated whether tumor-related seizures were associated with outcome. OS and RFS did not differ between patients with and without preoperative seizures (OS: undefined *versus* 124 months, *p* = 0.906; RFS: 56 *versus* 87 months, *p* = 0.639; Fig. 3C, D). This held true when only comparing patients with and without preoperative seizures in the subgroup of meningiomas WHO grade 2 and 3 with available TERT promotor mutation status (OS: 65 *versus* 124 months, *p* = 0.805; RFS: 56 *versus* 34 months, *p* = 0.670). Moreover, postoperative seizures were not associated with worse outcome (OS: 96.5 *versus* 124 months, *p* = 0.831; RFS: 96 *versus* 56 months, *p* = 0.524).

## Discussion

Tumor-related epilepsy is common in patients with intracranial meningioma. Different risk factors have been found to be associated with preoperative and postoperative seizures including age, peritumoral edema, tumor localization and brain invasion, thus impacting clinical patient management [8]. Nevertheless, studies investigating predictors for seizures in patients with meningioma WHO grade 2 and 3 in the context of the 2021 WHO classification are currently still missing. In particular, the impact of genetic alterations like TERT promotor mutations on tumor-related epilepsy remains unclear.

In this retrospective study, we reviewed 95 patients with meningioma WHO grade 2 and 3 according to the current WHO classification receiving microsurgical tumor resection. In our study population, age, sex distribution and histology were characteristic for meningiomas, and different tumor localizations were evenly distributed [18, 19, 22, 28]. The incidence of patients suffering from preoperative tumor-related seizures was 29.5%, which was in line with previously published series reporting rates of 13 to 40% [3, 8, 14]. Presence of edema and edema volume correlated with the development of preoperative seizures in univariate analyses matching previous reports including meningiomas WHO grade 1 [3, 12]. After adjusting for possible confounders in multivariate analyses, presence of edema remained the only independent risk factor with over sixfold odds to develop preoperative seizures. Notably, previously described additional risk factors including age, tumor localization and brain invasion on histology were not found to be of significance for tumor-related epilepsy in our patient cohort [3, 12]. However, one has to account for possible bias in these analyses due to small sample size. Overall survival and recurrence-free survival did not differ between patients presenting with or without tumor-related epilepsy. Interestingly, TERT promotor mutations status and diagnosis of a meningioma WHO grade 3 had no effect on the development of preoperative seizures. On a cautionary note, sample sizes of patients with histologically and molecularly more aggressive meningioma (WHO grade 3, TERT promotor mutated) were small, limiting the model’s usefulness in evaluating the effect of more aggressive meningiomas on the risk of preoperative seizures. As a key limitation, data on extended molecular diagnostics, next-generation sequencing or DNA methylation-based diagnostics were not available for all patients in this cohort. As such, the impact of different molecular alterations in molecularly well-defined meningiomas on tumor-related epilepsy could not be evaluated sufficiently and warrants further studies.

In regard to postoperative seizure control, tumor resection mitigated the risk for seizures drastically in our study cohort. In contrast to previously reported postoperative seizure freedom rates of about 70% [8], only two out of 28 patients with preoperative seizures developed postoperative seizures in our study cohort (seizure freedom rate of 92.9%). Of note, about one third of all postoperatively seizure-free patients received AEDs at the end of follow-up. Postoperative complications including hemorrhage at the surgical site, infections and hydrocephalus were shown to increase the risk for postoperative seizures and were seldomly encountered in the present study population [28]. On a cautionary note, follow-up time was limited in a subset of patients, limiting our findings on long-term postoperative seizure control. Furthermore, no particular risk factors for the development of postoperative seizures could be derived from our patient population. On a cautionary note, the sample size of patients suffering from postoperative tumor-related epilepsy was small and insufficient for detailed multivariate analyses. Previous studies have defined peritumoral edema on preoperative imaging, higher WHO grading and lower extent of resection as independent risk factors for unfavorable postoperative seizure outcome [22]. In this context, residual tumor is thought to continue irritation of the cerebral cortex, inducing epileptic discharges and seizures; however, our study has found no association between extent of resection and postoperative epilepsy. Importantly, follow-up imaging was insufficient for detailed volumetric analyses on postoperative edema in our patient cohort, limiting insight on the role of postoperative edema on the development of tumor-related seizures after surgery.

Pathogenetic mechanisms driving tumor-related epilepsy remain complex and include aberrant neuronal migration, changes in synaptic vesicles and glial gap-junction coupling alterations [6, 9, 20, 23]. On a cellular level, morphologic changes lead to a higher concentration of excitatory voltage-dependent Na^+^, Ca^++^ and glutamate receptors, diminishing inhibitory synapses and propagating excitatory influences leading to cortical hyperexcitability [5, 6]. Moreover, brain invasion has been shown to be associated with preoperative seizures, leading to a disruption of the pial-glial basement membrane, potentially inducing astrocytic responses and altering the tumor microenvironment similar to primarily intra-axial malignancies [5]. In our study cohort, brain invasion was not directly associated with preoperative seizures. However, patients with invasive tumors demonstrated higher edema volume, which in turn was predictive for tumor-related seizures. The tumor and its peritumoral edema microenvironment represent a peculiar epileptogenic milieu comprising physical and metabolic components, leading to local hypoxia, pH changes and excessive concentrations of glutamate and aspartic acid [14, 23]. Especially peritumoral edema has been demonstrated to be of importance in the development of tumor-related seizures. Vasogenic edema, stemming from an increased expression of the vascular endothelial growth factor (VEGF), alters the peritumoral cortex, thus affecting neurotransmitter pathways, again leading to cortical hyperexcitability [6, 20, 29]. Changes on a cellular and molecular level induced by the presence of perifocal edema could potentially trigger tumor-related epilepsy irrespective of absolute edema size. In line with this hypothesis, we did not find any linear correlation between edema volume and epilepsy in multivariate analyses. Overall, edema represents an epiphenomenon for different factors driving epileptogenesis as described above and serves as a surrogate marker on preoperative imaging. Apart from the peritumoral edema apparent on preoperative imaging, patients without edema were also observed to develop preoperative seizures in selected cases of our patient cohort. Here, slow-growing meningiomas might not induce perifocal edema, yet lead to cortical deafferentation by either mechanically or vascularly isolating brain regions, driving epileptogenesis [5, 9, 25].

Anticonvulsant treatment for tumor-related epilepsy remains challenging. Prior to surgery, prophylactic AED therapy in all patients presenting with intracranial meningioma is currently not recommended according to guidelines [4, 11], yet in a real-world setting, AEDs are often prescribed in patients with large convexity meningiomas especially presenting with perifocal edema [10]. In our series, patients received AEDs prophylactically in selected cases due to extensive tumor or peritumoral edema volume > 100 cm^3^ and localization at the convexity. Randomized-controlled trials investigating the benefit of prophylactic AEDs in meningioma surgery are warranted to provide class 1 evidence. Despite tumor resection reducing the risk for seizures significantly, postoperative seizure control is not achieved in a substantial subset of patients. Wirsching et al*.* proposed a clinical score (*STAMPE2*) to guide the indication for postoperative anticonvulsant therapy considering sensorimotor deficits, age, major surgical complications, history for preoperative seizures, epileptiform discharges on postoperative EEG, edema and tumor progression on follow-up imaging [28]. Importantly, resistance to commonly used AEDs confronts treating physicians with a difficult task and often requires inpatient diagnostic work-up and anticonvulsant therapy adjustment, monitoring for effectiveness, drug interactions and adverse events. Studies advancing pathophysiological insights into the development of tumor-related epilepsy are warranted and form the basis for new drug development.

Key limitations of our study include the limited sample size as well as different adjuvant treatment strategies in our patient cohort including watch-and-wait and adjuvant radiotherapy, possibly introducing bias. Large prospective studies are warranted to further characterize predictors for perioperative epilepsy in patients with meningioma WHO grade 2 and 3 undergoing tumor resection. Given the retrospective design of our study, no sample size calculations were performed a priori. Patients excluded due to missing clinical or imaging data could have introduced selection bias. Concerning peritumoral AED management, details on exact pharmacological treatment were limited due to the retrospective nature of our study.

## Conclusion

In conclusion, our data show that peritumoral edema is a strong risk factor for preoperative tumor-related seizures in patients with intracranial meningioma WHO grade 2 and 3 while molecular or histological features were not associated with tumor related epilepsy. Tumor resection was highly effective in achieving seizure freedom.

## Supplementary Information

Below is the link to the electronic supplementary material.

Supplementary file1 (DOCX 37 KB)

Supplementary file2 (DOCX 34 KB)

## Data Availability

The data presented in this study are available on request from the corresponding authors. The data are not publicly available due to the guidelines of the Institutional Review Board of the Ludwig-Maximilians-University in Munich.

## Abbreviations

CE: Contrast enhancing
CI: Confidence interval
CNS: Central nervous system
FLAIR: Fluid-attenuated inversion recovery
RANO: Response Assessment in Neuro-Oncology
ILAE: International League Against Epilepsy
IR: Interquartile ratio
MRI: Magnetic resonance imaging
OR: Odds ratio
OS: Overall survival
RFS: Recurrence-free survival
TERT: Telomerase reverse transcriptase
VEGF: Vascular endothelial growth factor
WHO: World Health Organization
