# Supplementary material for: Perifocal edema is a risk factor for preoperative seizures in patients with meningioma WHO grade 2 and 3
Source: Acta Neurochir (Wien). 2024 Apr 6;166(1):170. doi: 10.1007/s00701-024-06057-3 (PMC10998776; doi:10.1007/s00701-024-06057-3)
Supplement: Supplementary file 1 — Supplementary file1 (DOCX 37 KB) [file 701_2024_6057_MOESM1_ESM.docx]

**Supplementary Table 1: Patient characteristics**

|  |  | **Preoperative seizures** | **No preoperative seizures** | **Total** | ***p* value** |
| --- | --- | --- | --- | --- | --- |
| **Overall, n (%)** |  | 23 (34.3%) | 44 (65.7%) | 67 (100%) |  |
| **Age at diagnosis, mean (SD)** |  | 64.8 ± 11.9 | 59.5 ± 17.1 | 61.3 ± 15.6 | 0.185 |
| **Sex, n (%)** | *Female* | 15 (65.2%) | 28 (63.6%) | 43 (64.2%) | 0.999 |
|  | *Male* | 8 (34.8%) | 16 (36.4%) | 24 (35.8%) |  |
| **Clinical characteristics** | | | | | |
| **Additional symptoms, n (%)** | *Cranial nerve palsy* | 1 (4.3%) | 9 (20.5%) | 10 (14.9%) | ***0.001** |
|  | *Cognitive deficits* | 1 (4.3%) | 9 (20.5%) | 10 (14.9%) |  |
|  | *Headache* | 0 (0%) | 11 (25.0%) | 11 (16.4%) |  |
|  | *Sensorimotor deficits* | 4 (17.4%) | 6 (13.6%) | 10 (14.9%) |  |
|  | *Aphasia* | 2 (8.7%) | 0 (0%) | 2 (3.0%) |  |
|  | *Incidental* | 0 (0%) | 11 (25.0%) | 11 (16.4%) |  |
| **postOP (transient) deficits, n (%)** | *Yes* | 2 (7.1%) | 8 (18.2%) | 10 (14.9%) | 0.297 |
|  | *No* | 26 (92.9%) | 36 (81.8%) | 62 (92.5%) |  |
| **MRI characteristics** | | | | | |
| **Localization** | *Convexity* | 9 (39.1%) | 17 (38.6%) | 26 (38.8%) | 0.399 |
|  | *Skull base* | 10 (43.5%) | 16 (36.4%) | 26 (38.8%) |  |
|  | *Parafalcine* | 0 (0%) | 5 (11.4%) | 5 (7.5%) |  |
|  | *Intraventricular* | 4 (17.4%) | 6 (13.6%) | 10 (14.9%) |  |
| **Extent of resection, n (%)** | *Gross total resection* | 15 (65.2%) | 32 (72.7%) | 47 (70.1%) | 0.580 |
|  | *Subtotal resection* | 8 (34.8%) | 12 (27.3%) | 20 (29.9%) |  |
| **Contrast enhancement, n (%)** | *homogenous* | 14 (60.9%) | 20 (45.5%) | 34 (50.7%) | 0.305 |
|  | *heterogenous* | 9 (39.1%) | 24 (54.5%) | 33 (49.3%) |  |
| **Edema, n (%)** | *Present* | 21 (91.3%) | 26 (59.1%) | 47 (70.1%) | ***0.010** |
|  | *Absent* | 2 (8.7%) | 18 (40.9%) | 20 (29.9%) |  |
| **Volumetric analyses, median cm^3^ (IR)** | *Tumor volume* | 34 (15-58) | 27 (16-65) | 31 (16-62) | 0.551 |
|  | *Edema volume* | 53 (9-106) | 5 (0-38) | 14 (0-66) | ***0.002** |
|  | *Total volume* | 107(41-156) | 45 (18-128) | 69 (30-145) | ***0.011** |
| **Neuropathology** | | | | | |
| **WHO grade 2021, n (%)** | *Grade 2* | 21 (91.3%) | 37 (84.1%) | 58 (86.6%) | 0.708 |
|  | *Grade 3* | 2 (8.7%) | 7 (15.9%) | 9 (13.4%) |  |
| **TERT promotor, n (%)** | *Wildtype* | 22 (95.7%) | 39 (88.6%) | 61 (91.0%) | 0.656 |
|  | *Mutated* | 1 (4.3%) | 5 (11.4%) | 6 (9.0%) |  |
| **Brain invasion, n (%)** | *Present* | 10 (43.5%) | 11 (25.0%) | 21 (31.3%) | 0.167 |
|  | *Absent* | 13 (56.5 %) | 33 (75.0%) | 46 (68.7%) |  |
| **Adjuvant treatment** | | | | | |
| **Radiotherapy, n (%)** | *Yes* | 9 (39.1%) | 10 (22.7%) | 19 (28.4%) | 0.169 |
|  | *No* | 14 (60.9%) | 34 (77.3%) | 58 (86.6%) |  |

Characteristics are given for patients with intracranial meningioma WHO 2021 grade 2 or 3 with available TERT promotor mutation status (n = 67), patients presenting with preoperative seizures (n = 23) or without preoperative seizures (n = 44). *Abbreviations*: *n –* count; *postOP* – postoperative; *SD –* standard deviation. The significance level was set at *p* ≤ 0.05.
