# Supplementary material for: Perifocal edema is a risk factor for preoperative seizures in patients with meningioma WHO grade 2 and 3
Source: Acta Neurochir (Wien). 2024 Apr 6;166(1):170. doi: 10.1007/s00701-024-06057-3 (PMC10998776; doi:10.1007/s00701-024-06057-3)
Supplement: Supplementary file 2 — Supplementary file2 (DOCX 34 KB) [file 701_2024_6057_MOESM2_ESM.docx]

**Supplementary Table 2: Multivariate logistic regression analysis for predicting preoperative seizures**

Multivariate logistic regression for predicting preoperative seizures, adjusted for statistically significant and clinically meaningful risk factors in patients with intracranial meningioma WHO 2021 grade 2 or 3 and available TERT promotor mutation status. 5 patients with intraventricular localization were excluded due to multicollinearity (n = 62). The significance level was set at p ≤ 0.05. *Abbreviations:* *R –* reference.

| **Risk factor** | **Odds ratio** | **95% CI** | ***p* value** |
| --- | --- | --- | --- |
| **Age at diagnosis, years** | 1.04 | 0.99, 1.09 | 0.151 |
| **Sex** |  |  | 0.738 |
| *Female (R)* | 1 |  |  |
| *Male* | 1.29 | 0.28, 5.87 |  |
| **Location, n (%)** |  |  |  |
| *Skull base (R)* | 1 |  |  |
| *Convexity* | 1.55 | 0.35, 7.25 | 0.563 |
| *Parafalcine* | 2.81 | 0.43, 19.66 | 0.280 |
| **Edema** |  |  |  |
| *Absent (R)* | 1 |  |  |
| *Present* | **6.61** | **1.18, 58.12** | ***0.049** |
| **Edema volume, cm^3^** | 1.01 | 0.99, 1.02 | 0.312 |
| **Tumor volume, cm^3^** | 1.00 | 0.99, 1.01 | 0.604 |
| **WHO grade 2021** |  |  |  |
| *Grade 2 (R)* | 1 |  |  |
| *Grade 3* | 1.30 | 0.02, 80.86 | 0.899 |
| **TERT promotor** |  |  |  |
| *Wildtype (R)* | 1 |  |  |
| *Mutated* | 0.14 | 0.00, 13.35 | 0.378 |
| **Brain invasion, n (%)** |  |  |  |
| *Absent (R)* | 1 |  |  |
| *Present* | 1.24 | 0.26, 5.79 | 0.785 |
